# Ribosome decision graphs for the representation of eukaryotic RNA translation complexity

**DOI:** 10.1101/gr.278810.123

**Published:** 2024-04

**Authors:** Jack A.S. Tierney, Michał Świrski, Håkon Tjeldnes, Jonathan M. Mudge, Joanna Kufel, Nicola Whiffin, Eivind Valen, Pavel V. Baranov

**Affiliations:** 1 School of Biochemistry and Cell Biology, University College Cork, Cork T12 K8AF, Ireland;; 2 SFI Centre for Research Training in Genomics Data Science, University College Cork, Cork T12 K8AF, Ireland;; 3 Institute of Genetics and Biotechnology, Faculty of Biology, University of Warsaw, 02-106 Warsaw, Poland;; 4 Computational Biology Unit, Department of Informatics, University of Bergen, NO-5020 Bergen, Norway;; 5 European Molecular Biology Laboratory, European Bioinformatics Institute, Wellcome Genome Campus, Hinxton CB10 1SD, Cambridge, United Kingdom;; 6 The Big Data Institute and Wellcome Centre for Human Genetics, University of Oxford, Oxford OX3 7BN, United Kingdom;; 7 Department of Biosciences, University of Oslo, 0316 Oslo, Norway

## Abstract

The application of ribosome profiling has revealed an unexpected abundance of translation in addition to that responsible for the synthesis of previously annotated protein-coding regions. Multiple short sequences have been found to be translated within single RNA molecules, within both annotated protein-coding and noncoding regions. The biological significance of this translation is a matter of intensive investigation. However, current schematic or annotation-based representations of mRNA translation generally do not account for the apparent multitude of translated regions within the same molecules. They also do not take into account the stochasticity of the process that allows alternative translations of the same RNA molecules by different ribosomes. There is a need for formal representations of mRNA complexity that would enable the analysis of quantitative information on translation and more accurate models for predicting the phenotypic effects of genetic variants affecting translation. To address this, we developed a conceptually novel abstraction that we term ribosome decision graphs (RDGs). RDGs represent translation as multiple ribosome paths through untranslated and translated mRNA segments. We termed the latter “translons.” Nondeterministic events, such as initiation, reinitiation, selenocysteine insertion, or ribosomal frameshifting, are then represented as branching points. This representation allows for an adequate representation of eukaryotic translation complexity and focuses on locations critical for translation regulation. We show how RDGs can be used for depicting translated regions and for analyzing genetic variation and quantitative genome-wide data on translation for characterization of regulatory modulators of translation.

## Nascent need for abstract representation of mRNA decoding complexity

Until relatively recently, the available experimental evidence suggested that in eukaryotes each mRNA encoded only a single protein. Because only a single coding region was therefore expected to be translated, this region was conventionally termed the coding sequence (CDS). This view has been challenged by the development of the ribosome profiling technique, which enables the isolation and sequencing of RNA fragments protected by ribosomes and, hence, the detection of regions being translated (Ingolia et al. 2009). In essence, this technique is based on the capture of RNA fragments (footprints) within the ribosomes followed by their sequencing and mapping. Thus, it provides information on what sequences are being translated, whereas the densities of mapped footprints are indicative of the frequency with which ribosomes translate these sequences. Numerous ribosome profiling studies performed in cells from a variety of eukaryotes unexpectedly revealed abundant translation outside of CDS regions. This included the translation of short sequences in the supposedly untranslated regions (UTRs) of mRNAs, as well as in so-called noncoding RNAs, especially long noncoding RNAs (lncRNAs) (Ingolia et al. 2011; Michel et al. 2012; Ruiz-Orera et al. 2014; Andreev et al. 2015; Ji et al. 2015; Calviello et al. 2016; Johnstone et al. 2016; Chong et al. 2020; Chothani et al. 2022; Wright et al. 2022). These studies also showed the translation of N-terminally extended CDS regions owing to initiation at upstream non-AUG start codons (Fedorova et al. 2022) or C-terminally extended CDS regions owing to stop codon read-through (Dunn et al. 2013). A certain group of eukaryotic organisms (ciliates *Euplotes*) were found to use ribosomal frameshifting in thousands of their genes (Lobanov et al. 2017). Although most of these phenomena were first described before the advent of ribosome profiling (Baranov et al. 2002; Namy et al. 2004; Ivanov et al. 2010, 2011; Wethmar et al. 2010), they were considered rare. Certainly, very few cases have been cataloged by reference gene annotation projects, and no conventional abstraction has been developed to represent this translation complexity in annotations, schematic scientific diagrams, or analytical workflows. The lack of a formal framework for the representation of this complexity hampers our ability to generate accurate and biologically realistic annotations of translated sequences and to design mathematical models and computer simulations. In its absence, it is difficult or even impossible to quantitatively characterize multiple translation events and define their interrelationships.

To address this challenge, we developed a conceptually novel framework for abstract representation of translation complexity, which we term ribosome decision graphs (RDGs). RDGs solve many problems, such as the representation of multiple translated regions in the same mRNAs and alternative decoding mechanisms producing multiple proteoforms. We show how RDGs can be used for the accurate depiction of productive and nonproductive RNA translation (i.e., translation that does or does not lead to the production of a protein molecule), analysis of quantitative information on translation, and genetic variants affecting mRNA decoding.

## Representation of the complexity of mRNA translation using open reading frames leads to ambiguity

**Figure 1.**
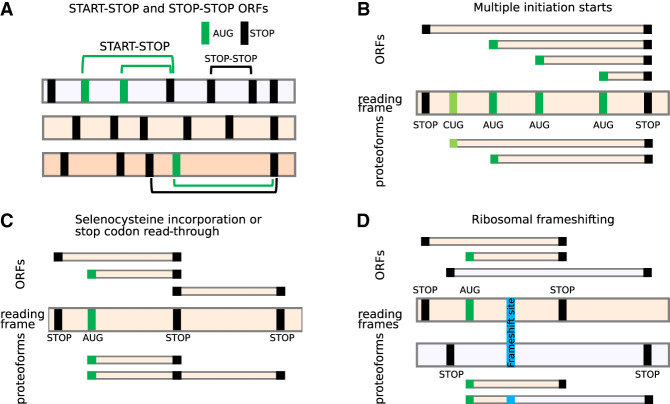
ORFs do not adequately represent translational complexity. (*A*) Two formal definitions of ORFs. Three reading frames are shown as horizontal bars, with vertical bars corresponding to AUG (green) or stop codons (black). Several examples of start-stop (green arcs) and stop-stop (black arcs) ORFs are shown. (*B*–*D*) The relationship between ORFs (*top*) and expressed proteoforms (*bottom*) for mRNAs with different locations of starts and stops (*middle*). Only two relevant reading frames are shown for simplicity. (*B*) An RNA encoding two proteoforms with alternative N termini owing to utilization of two start codons. Because of multiple potential AUG codons, there are many start-stop ORFs whose conceptual translation does not correspond to encoded proteoforms. A stop-stop ORF does not reflect the existence of alternative proteoforms. (*C*) In the case of stop codon read-through or selenocysteine insertion, ribosomes may read-through specific stop codons by incorporating an amino acid, yielding a product that cannot be described as a product of a single ORF. (*D*) Similarly, ribosomal frameshifting generates a *trans*-frame protein (blue) that does not represent a product of a single ORF.

The development of a conventional abstraction is undermined by the ambiguity of the terms used to define translated regions. For example, although translated regions are often described as open reading frames (ORFs) in literature or scientific discourse, gene annotation projects typically use only the term CDS, and only for regions considered to be protein-coding. Instead, an ORF would be regarded by implication as a potential translation that can be identified in silico. Here, we in effect consider three concepts in an attempt at unification: (1) that ORFs can be identified in silico whether or not they have evidence of translation; (2) that ORFs may undergo translation that does not lead to the production of a stable, functional protein; and (3) that ORFs that are known to be translated into proteins should alone be considered CDSs. In other words, most CDSs are ORFs, but not all ORFs are CDSs. In general, there are two definitions of ORFs, start to stop (start-stop) and stop to stop (stop-stop) (Sieber et al. 2018), as depicted in Figure 1A. Plotting the locations of potential start codons (usually AUGs) and stop codons in three reading frames is undoubtedly highly instrumental for examining potentially translated sequences. However, the common interpretation of nucleic acid sequences in terms of “translated ORFs” is superficial and frequently inaccurate and often leads to confusion as illustrated in Figure 1, B through D.

Perhaps the most frequent source of alternative translation in many eukaryotes, including humans, is the multiplicity of translation initiation sites. It arises predominantly from two common mechanisms involved in the selection of translation initiation sites: leaky scanning and reinitiation. *Leaky scanning* refers to the inefficient recognition of a start codon by the ribosome, resulting in the ribosome scanning complex scanning through the start codon and effectively ignoring it (Kozak 2002). Generally, ribosome scanning complexes assemble at the 5′ cap of RNAs and move along the transcript in the 3′ direction until they encounter a start site and initiate translation (Sonenberg and Hinnebusch 2009; Jackson et al. 2010; Hinnebusch 2014). However, recognition of start sites is a sequence-dependent stochastic process, in which usually only a proportion of scanning complexes finally initiate. Many factors play a role in determining the efficiency with which a ribosome initiates translation at a given codon. These include the identity of the codon and its surrounding sequence (known as the Kozak context) (Kozak 1987), as well as the dwell time of the scanning ribosome at that codon (Kozak 1989). Unless the combination of these factors is strictly optimal for initiation, at least a small fraction of scanning complexes will bypass the potential start site and continue scanning, allowing translation to be initiated further downstream. When a potential initiation site is a non-AUG codon or an AUG in a weak Kozak context, only a small proportion of scanning complexes will initiate translation. Thus, leaky scanning may result in the translation of different CDSs using numerous initiation sites, whereas initiation at start codons in the same reading frame can give rise to proteoforms with alternative N termini (PANTs) (Fig. 1B). A potentially large number of start codons may be used to initiate translation within the same stop-stop ORF, as is the case with the well-explored human *PTEN* gene, in which functionally distinct extended proteoforms are produced from multiple non-AUG starts (Tzani et al. 2016). Annotating all start-stop ORFs is problematic owing to the large number of potential start codons, and in certain cases, such as repeat-associated non-AUG (RAN) translation (Cleary and Ranum 2014; Nguyen et al. 2019), the exact position of the initiation site cannot even be easily identified.

In the case of stop codon read-through (Dunn et al. 2013; Loughran et al. 2014) and selenocysteine incorporation (Fig. 1C; Driscoll and Copeland 2003; Labunskyy et al. 2014), the translation products produced by these processes can be defined in computational terms as the fusion of an upstream start-stop ORF with a downstream stop-stop ORF. Gene annotation projects currently resolve these cases by “rewriting” the stop codon or the selenocysteine codon in the protein file, allowing them to code through. This results in the “extended ORF” that overlaps with a “standard ORF” in the same frame. For programmed ribosomal frameshifting (Fig. 1D), which is common in viruses but also infrequently occurs in cellular genes (Atkins et al. 2016), the description of translation using ORFs would require the introduction of the location of the frameshift site as both start and stop codon. This could enable the designation of the *trans*-frame protein product as a fusion of the two such “ORFs.” In practice, gene annotation may instead introduce an artificial indel modification of the natural DNA/RNA sequences to yield a single contiguous ORF; for example, the [T] corresponding to human hg38 assembly Chr 19: 2,271,440 nucleotide is deleted in both RefSeq (e.g., NM_004152.3) and Ensembl (e.g., ENST00000582888.8). To this end, existing gene annotation of in silico *trans*-frame translation may yield a protein sequence corresponding to the product generated in nature. However, it comes at the expense of producing an incorrect sequence of an mRNA molecule, which does not allow for the regulatory mechanism at play to be accurately represented.

The examples in Figure 1 are not exhaustive, and there are other translation phenomena that cannot be easily described using ORFs, such as translational bypassing (Herr et al. 2000; Nosek et al. 2015; Klimova et al. 2019) and StopGo (also known as StopCarryOn or 2A) (Atkins et al. 2007). Regardless of which ORF definition is used, the concept of a translated ORF is not adequate to represent the complexity of RNA translation.

## RNA translation is segmented

**Figure 2.**
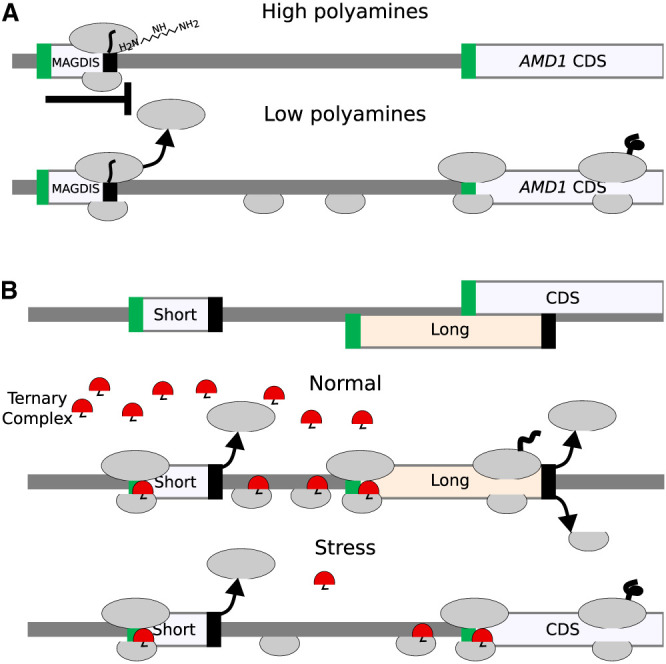
Relationship between translated segments within the same RNA. (*A*) A schematic of metabolite-dependent translation regulation via ribosome arrest at uORF, such as in *AMD1* mRNA. In the presence of a high concentration of polyamines, ribosomes with MAGDIS peptide stall at the end of the translon. (*B*) A schematic of delayed reinitiation mechanism enabling translation of selected mRNAs during global translation suppression caused by eIF2 complex phosphorylation during integrated stress response. When eIF2 is phosphorylated (specifically, serine 51 of its alpha subunit, encoded by *EIF2S1*), the concentration of the tRNAi*eIF2*GTP ternary complex (shown in red) decreases, and it requires a longer time and distance for the scanning ribosome complex to acquire it. As a result, the long uORF is bypassed, and initiation occurs at CDS.

Ribosome profiling has revealed the existence of a large number of short translated sequences, currently termed small or short ORFs (smORFs, sORFs) or Ribo-seq ORFs, as the term CDS is reserved for sequences encoding classical proteins (Mudge et al. 2022). Many Ribo-seq ORFs occur within the same RNA molecules. The lack of appropriate terminology reflecting the complexity of translation becomes even more evident when we consider the relationship between these translation segments. Upstream translation often influences downstream translation, and this dependency is known to be used to regulate gene expression. For instance, many short translated regions upstream of CDSs (termed upstream ORFs [uORFs]) have been found to regulate translation by blocking ribosomes via sensing-specific metabolites within the nascent peptide channel (Law et al. 2001; Rahmani et al. 2009; Laing et al. 2015; Ivanov et al. 2018; Hardy et al. 2019; for review, see Dever et al. 2023). This process is exemplified by translation regulation of the downstream CDSs by a short uORF in vertebrate *AMD1* encoding adenosylmethionine decarboxylase 1, a key enzyme in polyamine biosynthesis. The uORF encodes a short peptide MAGDIS that stalls ribosomes through its interactions with the ribosome in the presence of polyamines (Ruan et al. 1996). These stalled ribosomes prevent other ribosomes from binding and scanning downstream to initiate at *AMD1's* CDS. Thus, the uORF provides a negative feedback control mechanism for *AMD1* expression, inhibiting its synthesis when polyamine concentration is high but allowing for its synthesis when polyamine levels decrease (Fig. 2A).

In addition to leaky scanning, reinitiation is another process impacting start codon selection. Translation reinitiation occurs when small ribosomal subunits remain bound to the mRNA after translation is complete and reinitiate downstream from the terminating stop codon. This is thought to be common after the translation of short ORFs as it takes time for initiation factors to dissociate from the ribosome. In this way, the ribosome may remain capable of initiation after translating a small number of codons, although other factors are known to contribute to this process, allowing for reinitiation in some instances even after the translation of long ORFs. The detailed molecular mechanisms of these processes are described in dedicated reviews (Pestova et al. 2001; Kozak 2002; Sonenberg and Hinnebusch 2009; Jackson et al. 2010; Hinnebusch et al. 2016; Kearse and Wilusz 2017; Andreev et al. 2022). Reinitiation provides a platform for a rapid switch of gene expression on the translational level. Perhaps the most thoroughly studied is the case of delayed reinitiation (Hinnebusch 1997; Baird and Wek 2012; Andreev et al. 2023), which protects translation of certain mRNAs (e.g., human A*TF4*, yeast *GCN4*) from down-regulation during the integrated stress response (ISR) (Pakos-Zebrucka et al. 2016; Costa-Mattioli and Walter 2020). Under this condition, the reduced availability of the ternary complex (tRNAi* eIF2*GTP) increases the time required for postterminating ribosomes to bind the ternary complex enabling reinitiation. Therefore, the level of stress determines the location of the start codon at which reinitiation occurs. Figure 2B provides a schematic illustrating this mechanism.

It is unclear to what extent the translated products of such regulatory translation contribute to the functional cellular proteome beyond their potential contribution to the antigen pool, as many of them lack conservation at the protein level (Mudge et al. 2022; Prensner et al. 2023; Wacholder et al. 2023). Extreme cases of translation regulation without peptide synthesis are represented by minimal ORFs consisting of a start codon immediately followed by a stop codon. Although they obviously do not produce any functional peptide, some of them do have regulatory potential as strong ribosome stalling sites (Tanaka et al. 2016).

It is clear that translation complexity requires a unified and comprehensive abstraction that would adequately represent all translated regions—not only those that encode classical proteins—and reflect their mechanistic interrelationships. Such representation should be convenient to use by scientists when examining individual mRNA sequences and computer agents during programmatic analysis of large data sets.

## Ribosome paths

The complex nature of translational events and regulatory processes reveals the need to consider the entire passage of an individual ribosomal complex containing the same small ribosomal subunit along the mRNA, from the moment of preinitiation complex assembly at the 5′ cap (or IRES element) to the complete dissociation of both ribosomal subunits from the mRNA as a functional unit. We propose to term such a unit a ribosome path (RiboPath). It includes both regions that are scanned and those that are translated. As argued above, ORF is an inadequate descriptor of translated regions, and therefore, we want to define and assign a new, unambiguous name to an entity denoting *translated region* as encompassing the entire sequence of RNA translated by a fully assembled elongating ribosome from initiation codon through termination and dissociation of the large ribosomal subunit. We term this region *translon* (for the definitions of new terms, see Supplemental Table S1). It has already been suggested as a term specifying a unit of translation (Goel 1973) but has not yet been adopted. The main advantage of *translon* over ORF is that it is not constrained by the sequence (specific codons as boundaries). It is based on the process of translation and therefore may incorporate a variety of decoding mechanisms such as ribosomal frameshifting, stop codon read-through, translational bypassing, etc. (Baranov et al. 2002; Rodnina et al. 2020). The other term commonly used to indicate translated regions is *cistron*, for example, polycistronic or monocistronic mRNAs. However, this term was originally defined genetically; different cistrons should be responsible for different phenotypes; and it is being used inconsistently in the literature.

To simplify the introduction of the RiboPath concept, for now, we only consider initiation and reinitiation as the mechanisms producing alternative proteoforms. We will exclude other translation mechanisms. Nevertheless, our framework can easily be extended to incorporate other translation mechanisms as we discuss later.

**Figure 3.**
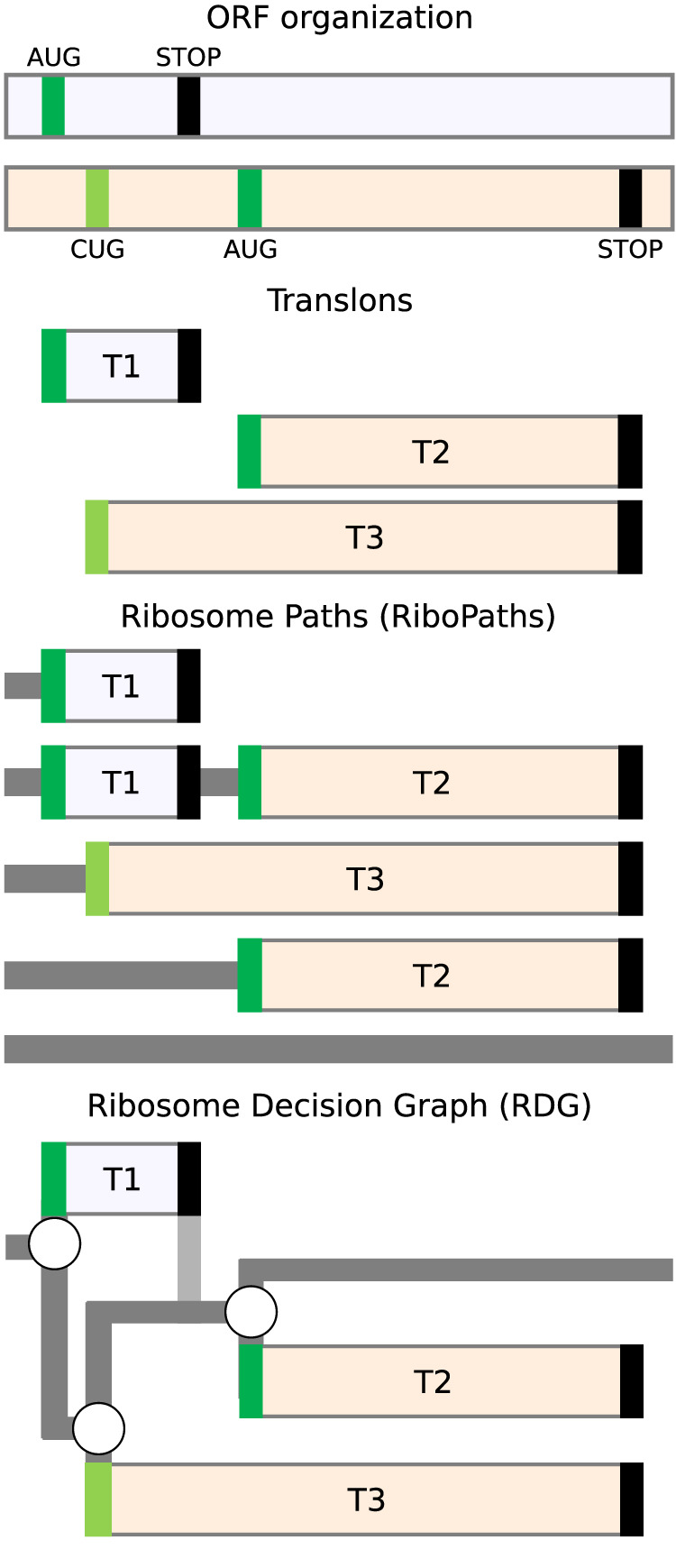
Ribosome paths through mRNA. (*Top*) An example of an RNA with three start codons (green for AUG and light green for CUG) located in two different reading frames depicted as differentially shaded horizontal bars. The translation of this mRNA is represented as a set of translons *below*; ribosome paths, further *below*; and ribosome decision graphs (RDGs), the *bottom*. RNA regions scanned by the ribosome are shown in dark gray; the vertical path in light gray represents the postterminating small ribosomal subunit that continues scanning and remains initiation-competent.

Figure 3 illustrates the RiboPath concept with an example of an mRNA encoding two proteoforms arising from alternative CUG and AUG initiation sites in one reading frame (cream) and a single upstream AUG codon in another frame (light lavender) as depicted in the ORF plot at the top. The corresponding translons are shown beneath. Alternative initiation and reinitiation allow the ribosome to pass through five different RiboPaths. The top RiboPath represents the ribosomes that initiate at the first AUG but fail to reinitiate further downstream, resulting in a path with a single translon T1. The second path corresponds to the ribosome that successfully reinitiates downstream, thus containing two translons, T1 and T2. In the third RiboPath, the ribosomes fail to initiate at the first AUG but start translation at the CUG, allowing for translon T3, which encodes an N-terminally extended proteoform relative to the product of translon T2. The fourth RiboPath corresponds to the ribosomes that fail to initiate at both the first AUG and the CUG but succeed at initiating at the second AUG so that its RiboPath consists of only one translon T2. Finally, the fifth RiboPath is unproductive and represents the ribosomes that have not initiated protein synthesis on this mRNA. The RiboPath presentation makes it clear that certain translons are mutually exclusive as they never occur on the same path; for example, a single ribosome cannot translate T1 and T3.

## Ribosome decision graphs

Once we represent the behavior of translating ribosomes in terms of paths, it is only natural to further represent these in terms of graphs (Fig. 3). The three initiation events in Figure 3 can be represented as branching points where the ribosome makes a “decision” of whether to initiate or not. We do not imply that ribosomes have free will; the decision is likely determined by the molecular composition and temporal thermodynamics of the local microstate. As in statistical mechanics, for practical purposes, it is appropriate to describe such decisions probabilistically, even if the underlying molecular processes are deterministic. The mRNA region engaged by ribosomes in Figure 3 can then be represented as a graph with three branching points. Stop codons in this graph are considered deterministic ends of translons as we exclude the possibility of stop codon read-through or reinitiation after long translons in our illustrative example. Following this notation, any translated RNA can be represented as a RDG. As in the representation of translation using ORFs/CDSs, RDGs may be either conceptual (representing potential) or real (e.g., experimentally supported). In the case of conceptual RDGs, all potential start codons in mRNAs could be used as branching points, for example, all AUGs, all CUGs, etc., depending on the specific parameters of the model. Such conceptual RDGs would be very complex graphs with a large number of branching points and possible paths. They are not suitable for evaluation by humans, but they provide a straightforward method for generating all theoretically possible products of RNA translation. This can be used for the subsequent mining of mass spectrometry data sets. A set of graphs with branching points sampled from the set of all possible branching points can be used to generate simulated ribosome profiling data. The comparison of simulated and real data would enable the determination of the best RDG fitting the experimental data, thus inferring the real branching points from the data. As exemplified further below, RDGs may also be useful for analyzing the impact of genetic variation, because variants that change or introduce new branching points (start and stop codons, frameshifts, etc.) would alter the RDG topology.

RDGs could also be used to annotate experimentally validated translations. In this case, only those translation events for which there is experimental evidence will be introduced as branching points. In most cases, these experimentally informed RDGs would be suitable for manual examination by researchers and would overcome the limitations of the data structures that are currently used for protein-coding annotation.

## Implementations of RDGs

**Figure 4.**
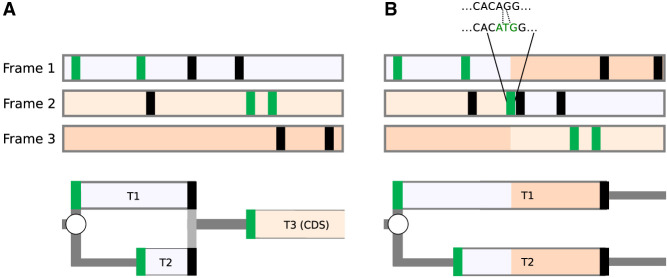
Representation of the effect of a genomic variant on CDS translation. Representations of the *NF2* mRNA for the reference sequence (*A*) and in the presence of the insertion variant (*B*). ORF organization is shown at the *top* with reading frames shaded differentially according to the reference sequence. AUG and stop codons are represented as green and black vertical bars, respectively. RDGs are shown at the *bottom*. Given the low probability of leaky scanning through the first two AUGs (see text), it is expected that the translon T3 corresponding to the CDS cannot be translated in the pathogenic variant sequence.

To illustrate how RDGs can be used to represent the impact of variation within 5′ leader sequences (i.e., 5′ UTR) on downstream translation, we selected the *NF2* variant responsible for neurofibromatosis type 2 (Whiffin et al. 2020). The 5′ leader sequence of the *NF2* mRNA contains an AUG start codon followed by an in-frame AUG codon in a strong Kozak context. This suggests that few (if any) ribosomes reach the CDS start via leaky scanning. It is far more likely that CDS translation involves reinitiation at the CDS start, as depicted in Figure 4A. A single-base insertion variant was identified in two unrelated individuals in a cohort of 1134 individuals diagnosed with neurofibromatosis type 2 (ENST00000338641:−66-65insT; GRCh37:Chr22:29999922A > AT) (Whiffin et al. 2020). This insertion causes both a shift in the reading frame and the introduction of another AUG. The shift extends translons T1 and T2, abrogating the initiation of translon T3 corresponding to the *NF2* CDS (Fig. 4B).

To illustrate how RDGs can be used for the representation of real translation data, we chose two simple examples, namely, human *NRAS* and *NXT1* mRNAs. The criteria for this selection were the existence of only a single transcript per gene according to GENCODE v.42 (Ivanov et al. 2018) and the ribosome profiling supporting translation of only a single AUG-initiated translon in addition to the annotated CDS. Of note, translation of most human 5′ mRNA leaders is more complex (see examples in Supplemental Fig. S1), and therefore, the advantages of using RDG representation for these are even greater but may not be suitable for introducing this concept as interpretation of ribosome profiling data is more difficult.

**Figure 5.**
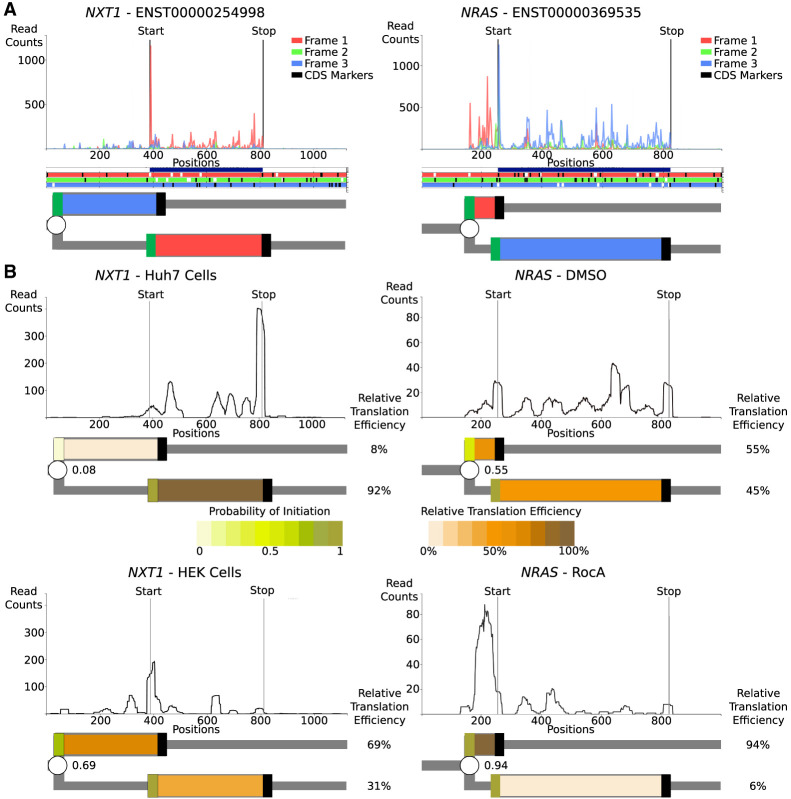
Representation of translation of human mRNAs. (*A*) The *top* plots are A-site subcodon footprint densities for *NXT1* and *NRAS* mRNAs colored to match the best-supported reading frame *below* in the ORF plots, in which AUGs are in white and stops are in black. Further *below* are RDG representations with translons colored to match the translated frame. The CDS starts are treated deterministically, and unproductive RiboPaths are not shown. (*B*) Densities of ribosome A-site footprints obtained from ribosome profiling under different conditions (*NRAS*) or from different cells (*NXT1*). RDGs are shown *below* each density plot, with translons colored as a heatmap reflecting relative translation efficiencies. Branching points (starts) are also colored as heatmaps, reflecting the inferred probability of their initiation. The probabilities of translation initiation are shown as fraction decimals, whereas the relative translation efficiencies of each path are shown as percentages.

Examination of ribosome profiling data in Trips-Viz (Fig. 5A; Kiniry et al. 2021) for *NXT1* mRNA reveals translation of an upstream region in the −1 frame (blue translon) relative to the CDS (red translon). Similarly, examination of Trips-Viz data indicates translation upstream and in the +1 reading frame (red translon) relative to the annotated *NRAS* CDS (blue translon). For simplicity, the CDS starts are not depicted as a branching point and are considered to be 100% efficient translation initiation sites. As the translated regions in both graphs are overlapping, it is clear that the simultaneous translation of both translons by the same ribosome cannot occur, at least in the absence of 3′ to 5′ scanning of postterminating ribosomes (Gould et al. 2014).

In addition to representing qualitative information, RDGs also enable a quantitative representation of translation regulation. Because of the leaky scanning mechanism of translation initiation, the efficiencies of each CDS's translation in these two examples directly depend on the efficiencies of the upstream starts; for example, if all ribosomes initiated at the upstream starts, no CDS translation would be observed. The relative translation efficiencies of translons can be used to calculate the probabilities of initiation at the upstream starts (Michel et al. 2014). These probabilities may vary between different conditions or across different cell types owing to a variety of mechanisms, such as global changes in the stringency of start recognition (Loughran et al. 2012; Fijałkowska et al. 2017) or specific regulation of mRNA via ribosome sensing of particular metabolites through interaction with the nascent peptide (Law et al. 2001; Rahmani et al. 2009; Laing et al. 2015; Ivanov et al. 2018; Hardy et al. 2019). Using RDG representations in this way makes it easier to characterize the relationship between translation events that are regulated (via changes in probabilities at branching points) and the relative rates of translons product synthesis.

To illustrate this with real examples, we examined the translation of the above genes using different ribosome profiling data sets (Fig. 5B). For *NRAS,* we used the data set from cells treated with rocaglamide A (RocA) and its untreated control (Iwasaki et al. 2016). As can be seen in Figure 5B, the silhouette of ribosome footprint density for the *NRAS* mRNA changes dramatically upon RocA treatment. These footprint densities can be used to calculate the relative translation efficiencies of *NRAS* translons and to derive the probability of translation initiation at the upstream starts (Supplemental Methods). By showing the relative synthesis rates and initiation probabilities as heatmaps, the relationship between these two translons becomes apparent. RocA treatment greatly increases translation initiation probability at the upstream start, most likely via the ability of RocA to clamp initiation factor EIF4A1 (previously known as EIF4A) to mRNAs containing specific sequence motifs (Iwasaki et al. 2016), which then reduces the downstream CDS translon. In the case of *NXT1*, we examined data obtained in two different cell lines, HeLa (Park et al. 2016) and Huh7 (Lintner et al. 2017). The silhouettes of ribosome footprint densities for *NXT1* mRNA are markedly different, as can be seen in Figure 5B. The RDG visualization of these differences in ribosome footprint densities pinpoints the upstream start as the pivotal element of cell-specific regulation of *NXT1* translation. For HeLa samples, the translation initiation at the upstream start is highly efficient, making the upstream start predominant. In contrast, for Huh7 samples, efficiency at this start is much lower, and consequently, the CDS translon is predominant. The reasons for these cell-specific differences are beyond the scope of this work, but several mechanisms may be responsible, including different levels of translation factors that recognize translation initiation starts (Anisimova et al. 2023).

RDG figures, such as those presented in this paper, are significantly time-consuming to produce manually. To show this concept without requiring the manual generation of RDGs, we also introduce a supplemental software, RDG-Viewer (available at https://colab.research.google.com/drive/1f5iSgy5DAXeq27Lx1fCyngm4IjinkgC5?usp=sharing). This Google Collaboratory notebook uses graph construction functionality from the RDG Python package (https://pypi.org/project/RDG/), which is currently under development (https://github.com/JackCurragh/RDG). For more details, please see the Supplemental Material.

**Figure 6.**
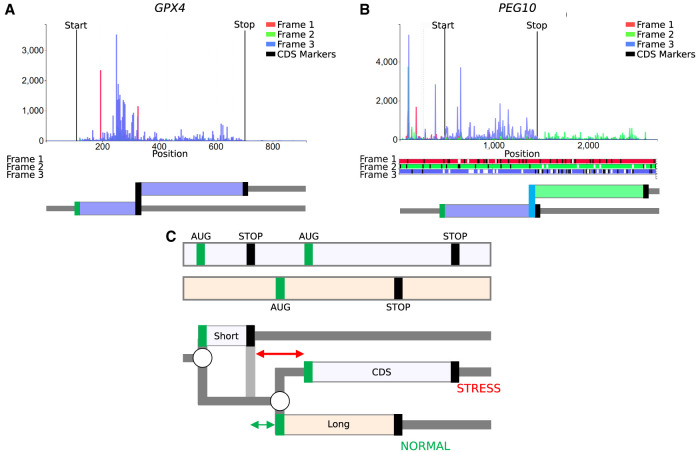
RDG representations of special cases. (*A*) mRNA encoding selenoprotein GPX4 and (*B*) *PEG10* mRNA requiring ribosomal frameshifting for its expression. Trips-Viz screenshots of ribosomal profiling density for these mRNAs are at the *top* with ORF plots *beneath* and RDG representations further *below*. (*C*, *top*) An ORF plot containing three translons (protein-coding CDS and two uORFs, short and long), which is a minimal requirement for the mechanism of delayed reinitiation. (*Below*) The corresponding RDG with green and red representing the predominant ribosome paths for normal and stress conditions, respectively. Arrows indicate the distance sufficient for scanning ribosomes to bind ternary complexes under normal (green) or stress (red) conditions.

One of the attractive features of the RDG concept is its expandability. In the RDG examples above, we limited branching points only to starts where initiation and reinitiation events can occur. The most basic information for generating RDGs that allows only leaky scanning would require only locations of starts in a transcript because in-frame stop codons are identifiable from the sequence and are treated deterministically as the ends of translons. However, the concept can be extended to incorporate annotations for any nondeterministic translation events, such as stop codon read-through or selenocysteine insertion (Figs. 1C, 6A), ribosomal frameshifting (Figs. 1D, 6B), translational bypassing (Herr et al. 2000), and even as-yet-undiscovered translation phenomena. Annotation schema in Supplemental Material provide an example of how such annotation could be organized computationally.

Despite the apparent simplicity of RDGs notations, it would be naive to expect that it can represent a full range of translational mechanisms. For example, in the case of a delayed reinitiation mechanism (Hinnebusch 1997; Baird and Wek 2012; Andreev et al. 2023) that makes the translation of certain mRNAs resistant to global down-regulation during the ISR, it is not sufficient to simply add a stop codon as a branching point, allowing either ribosome dissociation from mRNA or reinitiation downstream. This is because the reduced availability of the ternary complex (tRNAi*eIF2*GTP) increases the time required for the postterminating ribosomes to bind the ternary complex, thereby enabling reinitiation (Fig. 2B). Thus, it is not the probability of reinitiation, but the location of the start at which reinitiation will occur that changes during ISR. However, even in this case, the RDG concept can be useful to illustrate the mechanism, as shown in Figure 6C for a simplified mock transcript (for RDG for human *ATF4* that is regulated by delayed reinitiation, see Supplemental Fig. S1). It is conceivable to extend the concepts of RDGs with parameters linking scanning distance to reinitiation probability.

An important shortcoming of the presented solution is the difficulty of its application to genomic loci encoding multiple transcript isoforms. The purpose of RDGs is to represent molecular events that take place during the translation of a single mRNA molecule. Therefore, a single RDG can only be applied to a single mRNA sequence. However, the concept of representing biological sequences as graphs is gaining momentum with splice graphs for representing alternative splicing (Ryan et al. 2012) and variation graphs for representing pangenomes (Liao et al. 2023). Therefore, we envision that the RDG concept will fit into the emerging bioinformatic infrastructure of hierarchical representation of biological sequences as graphs, from genome to transcriptome to translatome.

## Conclusion

The RDG concept has the potential to significantly impact the study of RNA translation complexity. RDGs, in combination with Ribo-seq data, may shift the focus of differential translation analysis from changes in translation efficiencies of individual coding regions to the changes in the efficiencies of events regulating their translation. This focus shift will facilitate a mechanistic understanding of RNA translation regulation. Correlating mRNA translation with properties of RDGs (e.g., topology) may open a new possibility for identifying novel common mechanisms of translation regulation. In combination with information on genomic variants, RDGs have the potential to be instrumental in their phenotypic interpretation. Comparison of RDGs across orthologs would allow investigation of evolutionary constraints shaping translation regulation of specific genes.

## Supplementary Material

Supplement 1
