# Supplementary material for: Ribosome decision graphs for the representation of eukaryotic RNA translation complexity
Source: Genome Res. 2024 Apr;34(4):530–8. doi: 10.1101/gr.278810.123 (PMC11146595; doi:10.1101/gr.278810.123)
Supplement: Supplement 1 [file Supplemental_Material.pdf]

Supplemental Material

for

Ribosome Decision Graphs for the Representation of Eukaryotic RNA Translation Complexity

by

Jack A. S. Tierney[1,2,#], Michał Świrski[3,#], Håkon Tjeldnes[1,4], Jonathan M. Mudge[5], Joanna Kufel[3], Nicola Whiffin[6], Eivind Valen[4,7], Pavel V. Baranov[1].

1. Glossary of Concepts 2
2. RDG-Viewer 3
3. Complex Examples Visualised 4
4. Annotation Schema 5
5. Methods 6
6. References 7

1. Glossary of Concepts

A number of terms/concepts are introduced in this work. Supplemental Table S1 endeavours to delineate these terms and provide a clear reference resource for those wishing to build on these concepts.

Supplemental Table S1: Glossary of RDG Terms

Term	Definition	Notes
**Translon**	A Translon is the basic unit of translation. It refers to the entire path of a fully assembled elongating ribosome from initiation codon through termination and dissociation of the large ribosomal subunit.	Differs from the term Open Reading Frame (ORF) as it is not constrained by nucleotide sequence. It is instead only concerned with the activity of 80S ribosomes.
**Ribosome Path (RiboPath)**	A Ribosome Path refers to the way in which an individual ribosomal complex, containing the same small ribosomal subunit, traverses the mRNA from the moment of pre-initiation complex assembly at the 5’ cap to the complete dissociation of both ribosomal subunits from the mRNA.	A RiboPath consists of one or more translons joined by untranslated regions scanned by a ribosomal complex with the same 40S subunit.
**Ribosome Decision Graph (RDG)**	A Ribosome Decision Graph is a graph representing all RiboPaths pertaining to a single mRNA molecule.	A Ribosome Decision Graph is a graph based representation of the different ways how a specific RNA molecule can be translated. Nodes in these graphs represent the locations of alternative, nondeterministic events. This includes switches between unproductive (e.g. scanning) and productive (elongation) modes of translation as well as switches between reading frames (i.e. during ribosomal frameshifting) as well as yet undiscovered translation phenomena

2. RDG-Viewer

The RDG-Viewer is a Python 3 code that is a part of the RGD package (currently under development) that enables the generation and visualisation of RDGs. Specifically, it enables the following functionalities:

- Generate RDG using annotated translon coordinates as input.
- Generate RDG using the transcript nucleotide sequence and customizable parameters of translation as input.
- Generate images of the plots containing RDGs along with all translons grouped by three different reading frames. Supplemental Figure S1 shows two examples of such plots.

The nucleotide sequences could also be automatically obtained with gget (Luebbert and Pachter 2023) using transcript ENSEMBL ID or gene name symbols, in which case the first transcript isoform is selected from the list of ENSEMBL isoforms for that gene. The gene functionality is currently limited to *H. sapiens*.

The source code of RDG viewer along with its documentation is available at GitHub: https://github.com/JackCurragh/RDG-Viewer

The RDG-Viewer is also implemented in a Google Collaboratory notebook at https://colab.research.google.com/drive/1f5iSgy5DAXeq27Lx1fCyngm4IjinkgC5?usp=sharing and can be used for building RDGs and their visualisations for individual transcripts without a need of installing RDG package scripts locally.

3. Complex Examples Visualised

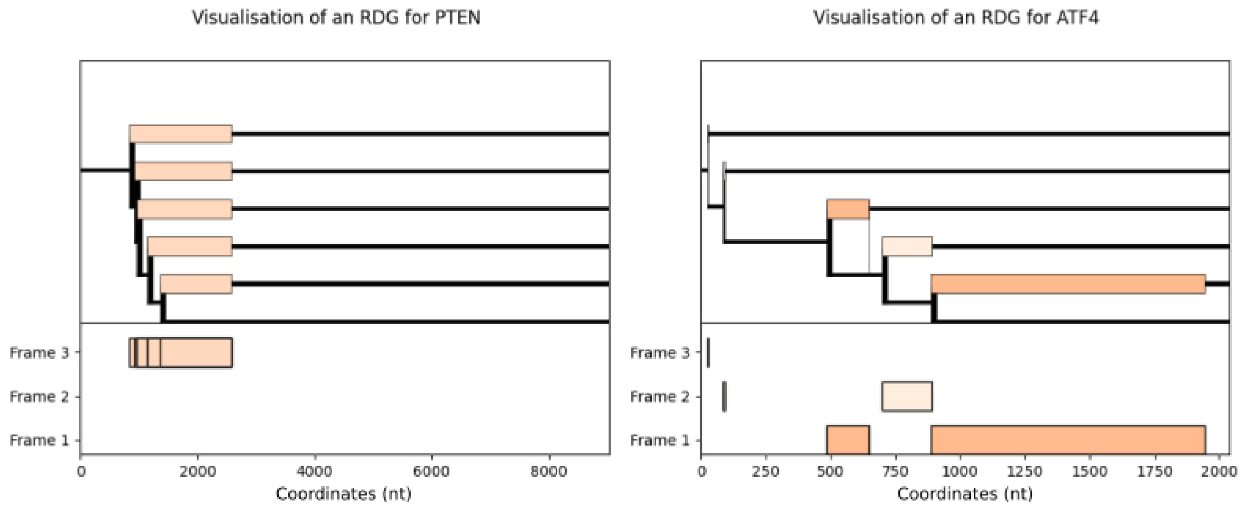

Supplemental Figure S1: RDG-Viewer plots. Left: Human *PTEN* transcript (ENST0000037195) with RDG constructed based on the data from (Tzani et al. 2016). **Right**: Human *ATF4* transcript (ENST00000337304) constructed by using all upstream AUGs as branching points*.* The upper part of these plots contain RDGs, while the bottom part shows translations grouped by three different reading frames.

4. Annotation Schema

The most basic information for generating RDGs requires only locations of starts in a transcript because in-frame stop codons are identifiable from the sequence and are treated deterministically as the ends of translons. Therefore, the example shown in Figure 1B of the main manuscript can be represented as a short array:

starts(x_1;x_2;x_3;x_4)

while the example in Figure 3 of the main manuscript as

starts(x_1;x_2;x_3)

However, the concept can be extended to incorporate annotations for any non-deterministic translation events. For example, to capture such phenomena as stop codon readthrough or selenocysteine insertion (Figures 1C and 6A in the main manuscript), an addition of a new type of branching point would be necessary: a stop codon, at which the ribosome could either terminate or incorporate an amino acid and continue translation.

starts(x_1); secs(y_1)

A frameshifting site would be yet another type of branching point where the ribosome could either continue translation in the same frame or shift to one of the alternative reading frames. For example, the case of ribosomal frameshifting (Figures 1D and 6B in the main manuscript) can be represented as

starts(x_1); shifts(i_1,s_1)

where shifts need to be assigned two coordinates, the coordinate of the last codon in the initial frame i_1 and the coordinate of the first codon in the shifted frame ***s***. Accordingly, $s_1 – i_1$ = 1 would correspond to +1 frameshifting, $s_1 – iy_1$ = -1 to -1 frameshifting, and so on. This notation is compatible not only with common types of frameshifting but also with extreme events such as bypassing in bacteriophage T4 gene *60* (34), where 50 nucleotides are skipped by the ribosome without translation, i.e. expressed as ***shifts(i,s)*** where ***s*** – ***i*** = 50.

Algorithms interpreting such annotations will be backward and forward-compatible since older algorithms could simply ignore new types of branching points.]

5. Methods

We used GENCODE v42 (Frankish et al. 2023) as a source of transcriptome annotation when searching for suitable loci and GENCODE v25 when producing visualisations in Trips-Viz (Kiniry et al. 2021). Pre-processed alignments from datasets GSE70211, GSE79664, and GSE94454 obtained in relevant studies (Iwasaki et al. 2016; Park et al. 2016; Lintner et al. 2017) were obtained from Trips-Viz. The translation efficiencies of examined regions were calculated as the number of ribosome footprints uniquely aligning to individual translons normalised over the length of translons used for the mapping. Five first codons of translons were excluded from mapping to avoid the distortions introduced by the high peaks at the starts. The probability of translation initiation was then calculated as $p=R_u/(R_u+R_d)$, where R_u and R_d are translation efficiencies of the upstream and downstream translons.

6. References

Frankish A, Carbonell-Sala S, Diekhans M, Jungreis I, Loveland JE, Mudge JM, Sisu C, Wright JC, Arnan C, Barnes I, et al. 2023. GENCODE: reference annotation for the human and mouse genomes in 2023. *Nucleic Acids Res* **51**: D942–D949.

Iwasaki S, Floor SN, Ingolia NT. 2016. Rocaglates convert DEAD-box protein eIF4A into a sequence-selective translational repressor. *Nature* **534**: 558–561.

Kiniry SJ, Judge CE, Michel AM, Baranov PV. 2021. Trips-Viz: an environment for the analysis of public and user-generated ribosome profiling data. *Nucleic Acids Res* **49**: W662–W670.

Lintner NG, McClure KF, Petersen D, Londregan AT, Piotrowski DW, Wei L, Xiao J, Bolt M, Loria PM, Maguire B, et al. 2017. Selective stalling of human translation through small-molecule engagement of the ribosome nascent chain. *PLoS Biol* **15**: e2001882.

Luebbert L, Pachter L. 2023. Efficient querying of genomic reference databases with *gget* ed. A. Valencia. *Bioinformatics* **39**: btac836.

Park J-E, Yi H, Kim Y, Chang H, Kim VN. 2016. Regulation of Poly(A) Tail and Translation during the Somatic Cell Cycle. *Mol Cell* **62**: 462–471.

Tzani I, Ivanov IP, Andreev DE, Dmitriev RI, Dean KA, Baranov PV, Atkins JF, Loughran G. 2016. Systematic analysis of the PTEN 5' leader identifies a major AUU initiated proteoform. *Open Biol* **6**: 150203.
